# Particle Size Inversion from Spectrally Resolved Full-Field Forward Scattering

**DOI:** 10.1021/acs.analchem.3c03178

**Published:** 2023-10-17

**Authors:** Miguel A. Báez-Chorro, Borja Vidal

**Affiliations:** Nanophotonics Technology Center, Universitat Politècnica de València, Valencia 46022, Spain

## Abstract

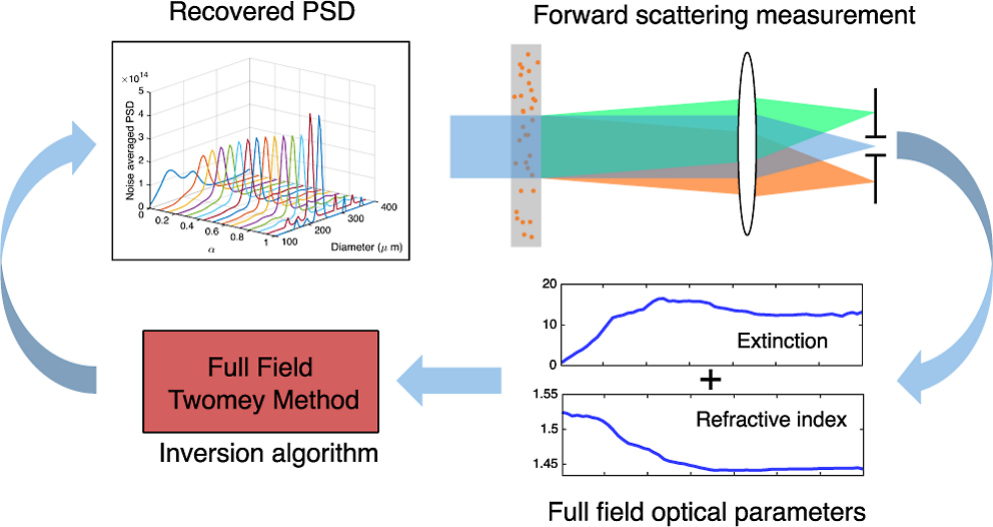

Ensemble particle sizing has traditionally relied on inversion of extinction measurements for the characterization of the particle size distribution (PSD) in particulate media. However, particulate media induce complex phase changes that contain valuable information about their structure. Here, we propose the use of coherent detection to derive particle size distributions in inhomogeneous samples from light scattering. This is achieved by exploiting THz waves, which allow for both extinction and refractive index information to be directly retrieved. A modified version of the iterative Twomey method is presented in order to take into account this information. Additionally, by using a forward model based on the Waterman–Truell formula for the complex refractive index, samples with absorption in both the matrix medium and the particulate phase can be measured. The inversion needs neither a priori assumptions nor constraints regarding the PSD shape. Numerical simulations show that this full-field approach reduces the error of the inversion process potentially up to 65% compared with inversion using only extinction data. Experimental validation of the technique is provided by measuring calibrated spherical glass particles inside a PTFE matrix and retrieving the PSD in the case of monodisperse and polydisperse samples showing an enhancement of up to 32% in comparison to inversion from extinction data.

## Introduction

Particle sizing using forward scattering measurements is a widespread tool in analytical chemistry with applications in the pharmaceutical and dairy industries, water management,^1^ immunoassays, as well as material and atmospheric sciences.^2−4^ Traditional electromagnetic sizing instruments typically work in the UV–vis–NIR. For example, forward scattering spectroscopy or turbidity^5,6^ is based on measuring the light extinction in the forward direction produced by the particles. This is realized over a range of wavelengths from UV to NIR and then Mie theory of scattering is used to fit a model depending on the size distribution to the extinction data.

Angularly resolved techniques, usually called light diffraction methods,^7^ are based on fitting extinction of light from monochromatic sources measured at multiple angular position (small angle, wide angle, or backscattering) to Mie theory or Fraunhofer diffraction approximation. Recently, this technique has evolved to be able to detect particle shape^8^ and is widely used for characterization of a wide range of sizes and materials.

Dinamic light scattering focuses on measuring the correlation of intensity fluctuations that particle produces when moving in front of a monochromatic light source as a result of its Brownian movement through the dispersing medium. This technique achieves the lowest range of sizes reaching into subnanometer suspensions, being suitable for the characterization of macromolecules and biological tissue.^9^

Other methods like optical shadowgraph work by capturing images of the dispersed particles and applying image processing to segment, count, and size them.^10^ These methods acquire quite accurate information about the particles since they do not make ensemble assumptions. However, they can be time-consuming since low flow speeds are needed for good image quality, and image processing can be computationally intensive. In this line, machine learning methods are being developed in order to speed this process.^11^

All of the previous methods are intensity-based. However, the presence of scattering centers modifies the full complex propagation constant of the medium, thus altering the phase, as well as the amplitude of the incident light. In the simplest case, a single small particle in a focused beam produces a phase shift as a consequence of the van Cittert–Zernicke theorem. This has been used by different interferometric schemes that allow the characterization of single particles purely from the phase information.^12,13^ However, conventional instruments aimed at retrieving the particle size distribution (PSD) of an ensemble of particles are blind to phase information, which could be combined with extinction information to enhance quality control systems.

The use of coherent detection is commonplace for pulsed ultrasound spectroscopy,^14−19^ where amplitude and phase information is readily available. However, particle sizing is usually performed using extinction data because of the high dependency of acoustic velocity on material properties.^19^ This and the fact that ultrasound transducers need contact with the sample limit its applicability.

A new path to extract particle size information can be developed by combining amplitude and phase information from the electromagnetic scattered field. A natural region to test this hypothesis is the THz band since many common THz techniques, such as THz time domain spectroscopy (THz-TDS),^20−25^ perform coherent measurements of the electric field and, like other optical approaches, allows noncontact characterization. Additionally, THz waves offer some interesting features on their own. THz instruments typically provide five-octave bandwidths, enabling enhanced precision and a wider range of particle sizes in the inversion as well as the capability to reach a range of particle sizes that are unattainable for lower wavelengths. Furthermore, the THz region opens particle sizing to new fields, such as polymer composites and other optically opaque materials.

Research on the interaction between THz waves and particulates has been mainly devoted to study and model the macroscopic effects of scattering by inhomogeneous media on the THz spectra. The attenuation induced by a medium formed by polyethylene (PE) pellets has been simulated^26^ using multiple scattering theory-based quasi-crystalline approximation (QCA). Later, a similar approach was taken but using instead the Waterman–Truell (WT) model for multiple scattering in conjunction with an iterative method.^27^ This theory, despite having a lower predictive power than QCA,^28^ gives a closed-form solution for the complex propagation constant instead of an implicit solution that has to be numerically solved, thus being computationally more efficient. Other researchers^29^ reviewed the possibilities of THz radiation for probing the packing structure of granular media through angle-resolved scattering at a single frequency with a phenomenological approach that was not aimed at extracting quantitative information about the size of the grains. Soon after, a study^30^ characterized mean diameters and polydispersity indexes of particulates from THz extinction spectroscopy and assuming Gaussian distributions. However, to the best of our knowledge, no previous work has tackled the retrieval of particulate information from coherent electromagnetic radiation or derived particle size distributions from THz waves.

In this work, the feasibility of using full-field electromagnetic radiation in the THz region to obtain information about the scattering behavior of the samples at many values of the scattering parameter is demonstrated. Unlike with most conventional approaches, this information can be obtained from solid samples with static particles, provided that the number of scatter centers in the illuminated region is statistically representative. To allow full-field particle sizing, a modification of the Twomey method (TM)^31^ for iterative inversion that combines data from both the extinction and the refractive index of the sample is presented. It is shown that the inclusion of refractive index data improves the accuracy of the retrieved PSD especially in the presence of noise.

This new paradigm can extend the use of particle size quality control to new sectors as the pharmaceutical industry, where the sizes of relevant particulate compounds used in drug tablets are in the range of THz wavelengths,^32^ as well as in the polymer and foam industries. Suitable samples include also optically opaque media and even matrix media with moderate absorption at the measuring frequencies.

## Experimental Section

### Full-Field Forward Model

Optical particle sizing is usually performed by measuring the extinction of a collimated beam passing through the test medium at several frequencies.^5^ The Fredholm integral shown in eq 1 relates the experimental measurement τ with the extinction efficiency *Q*_ext_ for a given frequency ν and particle diameter *D*, which can be computed using several scattering models, the normalized PSD, *f*(*D*) and the number density of particles, *N*_0_.
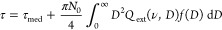
1

The second term in the right-hand side is used in aerosol and water turbidity inversion where the medium can be assumed to be lossless and tenuous. However, composite materials have matrix media with non-negligible absorption in the THz region, and this information needs to be accounted for. An additional term, τ_med_, is thus included in the extinction model to account for the extinction of the matrix.^33^

In order to take advantage of phase information, a more convenient model would be one that predicted the complex propagation constant of the heterogeneous medium. In the literature, mainly three models for the macroscopic propagation constant of a medium containing inclusions are considered. These are, in order of increasing accuracy, Foldy, Waterman–Truell (WT), and QCA with the Perkus–Yevick function (QCA-PY).

The first two models are developments based on the effective field approximation (EFA) of multiple scattering and give rise to the following closed form equations for the complex refractive index, *ñ*_eff_
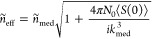
2
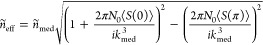
3where
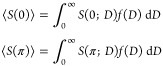
4and *k*_med_ = 2πν*ñ*_med_/*c*, being *c* the vacuum speed of light and *ñ*_eff_ and *ñ*_med_ the complex refractive indexes of the heterogeneous sample and the medium, respectively. These equations depend only on the propagation constant of the matrix medium where the particles are embedded and the Mie scattering functions for the species of particles sizes present in the medium. In the case of a distribution of particle sizes, each equation is easily transformed to accommodate an integral over all of the particle sizes, which can, in turn, be discretized via a quadrature decomposition of said integral.

Equation 2 corresponds to the multiple scattering model developed by Foldy^34^ and Lax.^35^ Their works consisted of obtaining the mean field resulting from the scattering contributions of the aggregate by performing configurational averages over random arrays of isotropic scatterers. In a later development, Waterman and Truell^36^ considered a general solution for any kind of spherical scatterer and obtained a governing equation for the propagation constant that can be seen in eq 3. This equation considers the forward and backward flows of scattered energy, represented by *S*(0) and *S*(π), which makes the WT model suitable for a greater range of size parameters.

**Figure 1:**
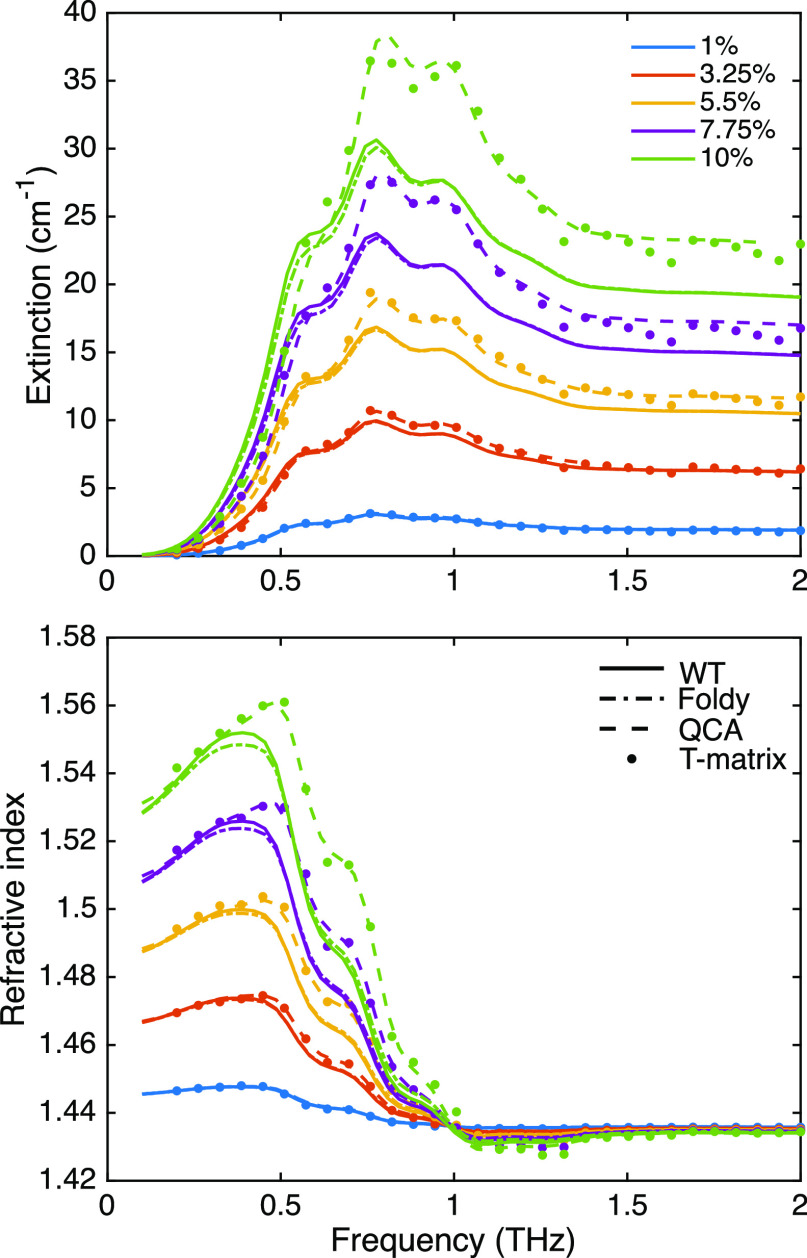
Simulation of optical constants with different multiple scattering approximations and different volume fractions. The particle size is set to 200 μm and the relative refractive index is 1.88 + *i*0.24 × ν[THz].

Later, Tsang and Kong^37^ developed a model suited for higher concentrations of scatterers based on the QCA with the Perkus–Yevick approximation for the pair distribution function of particle positions. The general result of this computation is an implicit system of equations for the propagation constant of the coherent wave through the media.^37,38^ A comparison of the mentioned scattering approximations can be seen in Figure 1, where the extinction coefficient and refractive index of a material containing spherical inclusions with a diameter of 200 μm are plotted against frequency for different volume fractions. In addition to the discussed analytical theories, the same kind of medium was simulated by using a more realistic full field simulation. The software used for that was the suit CELES,^39^ a CUDA accelerated MATLAB implementation of the multiparticle T-matrix method. This code allows for the computation of the electric field generated by a chosen ensemble of particles with given sizes, refractive indexes, and positions. The final result is the summation of the contribution of all the particles, which includes interparticle effects and multiple scattering at a given distance and a given wavelength. QCA theory is the closest match to the T-matrix simulations, while the other multiple scattering approximations are valid only for smaller volume fractions.

In this work, however, we employ the WT formula mainly for two reasons: first, stability and efficiency of the code implementation (stemming from the relative complexity of QCA dispersion relations vs Mie scattering functions); and second, due to the simplicity of the equations, which can be more easily integrated with the inversion algorithm that needs to take into account not only the extinction but also the refractive index. This choice limits our experimental applications to a volume fraction of around 5%.

### Iterative Inversion

When solving for the size distribution of the particulate, one has to solve eq 1 and find *f*(*D*) for a finite set of discretized diameters (*D*_*j*_, *j* = 1, ..., *M*) from the knowledge of the measured extinction in a discrete set of frequencies (ν_*i*_, *i* = 1, ..., *N*). This constitutes the problem of the inversion of a Fredholm integral equation of the first kind, which is a type of problem well-known in the remote sensing and particle sizing literature.^5,40,41^ In order to solve this, one has to first transform the continuous integral equation from eq 1 system of linear equations of the following form
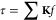
5where **K** constitutes a matrix containing the quadrature decomposition of the kernel integral that depends on the variables of τ and *f*, in this case, frequency (ν) and diameter (*D*).

This system of equations could be theoretically solved by inversion of matrix **K** or a least-squares approach can be taken to find an optimal solution. For the former, even assuming that the matrix **K** has an inverse, experimental errors and inhomogeneities arising from the quadrature decomposition make this matrix badly conditioned, and a stable solution cannot, in general, be found.

Conventional practice in inversion of extinction data calls for regularized inversion of quadrature decomposition. Tikhonov-like approaches are reliable ways of regularized inversion^5,42^ and a closed solution for the PSD can be found in terms of a free regularization parameter that needs to be adjusted. Many other inversion techniques are available for this task, and we refer to the recent review by Świrniak and Mroczka^43^ for a comprehensive comparative of inversion methods. However, conventional regularization is not suited for problems where the measurable quantity and the quadrature kernel are not linearly related, as in eq 5. This would be the case when trying to invert a problem such as that of eq 3.

In this work, we make use of another inversion approach based on iterative methods. These methods work by iteratively multiplying an ansatz PSD by an envelope factor that leverages both the similarity of the calculated parameters through the forward model and the shape of the scattering kernel. They are best suited for problems where the forward model kernel and the measured quantity are not linearly related and can be adapted to accept multiple sets of data. Besides, because of its multiplicative nature, iterative inversion methods automatically ensure non-negativity if the initial solution and the iterative factor are always positive. In particular, we will make use of an iterative algorithm devised by Twomey^44^ that has been applied for inversion problems with different kernel shapes.^45^ Twomey’s iterative method has the following formula
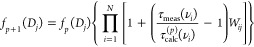
6where, for each iteration, the previous solution *f*_*p*_(*D*_*j*_) is multiplied by a factor that depends on the measured extinction τ_meas_, the extinction calculated though the forward model in eq 1 and adding the extinction of the matrix medium. *W*_*ij*_ acts as a weighting matrix and has the shape of an extinction kernel.

We adapted this iterative factor to include both extinction and refractive index information. Equation 7 shows the new iterative factor
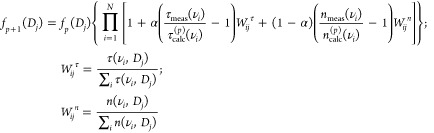
7where α is a parameter that weights the real and imaginary parts of the complex optical constant. This time, τ_calc_ = 4πν/(*c* Im{*ñ*_eff_}) and *n*_calc_ = Re{*ñ*_eff_} with *ñ*_eff_ obtained from eq 3.

Equation 7 includes an iterative factor involving both the refractive index and extinction. Henceforth, we will call it the full-field Twomey Method (FFTM). In this new formula, the smoothing kernels for each term need to be related with the shape of each data set. For this, we calculate the quadrature decomposition of the scattering functions *S*(0) and *S*(π) following^40^ for a range of values of ν and *D*. Then, the extinction coefficient and refractive index kernels (*W*_*ij*_^τ^ and *W*_*ij*_^*n*^) are obtained following eq 7. The τ and *n* employed to compute the values of *W*_*ij*_^τ^ and *W*_*ij*_^*n*^ differ from τ_calc_ and *n*_calc_ in that the former are not calculated using the integrals of eq 4 weighted by a PSD, but they are computed at individual diameter values.

**Figure 2:**
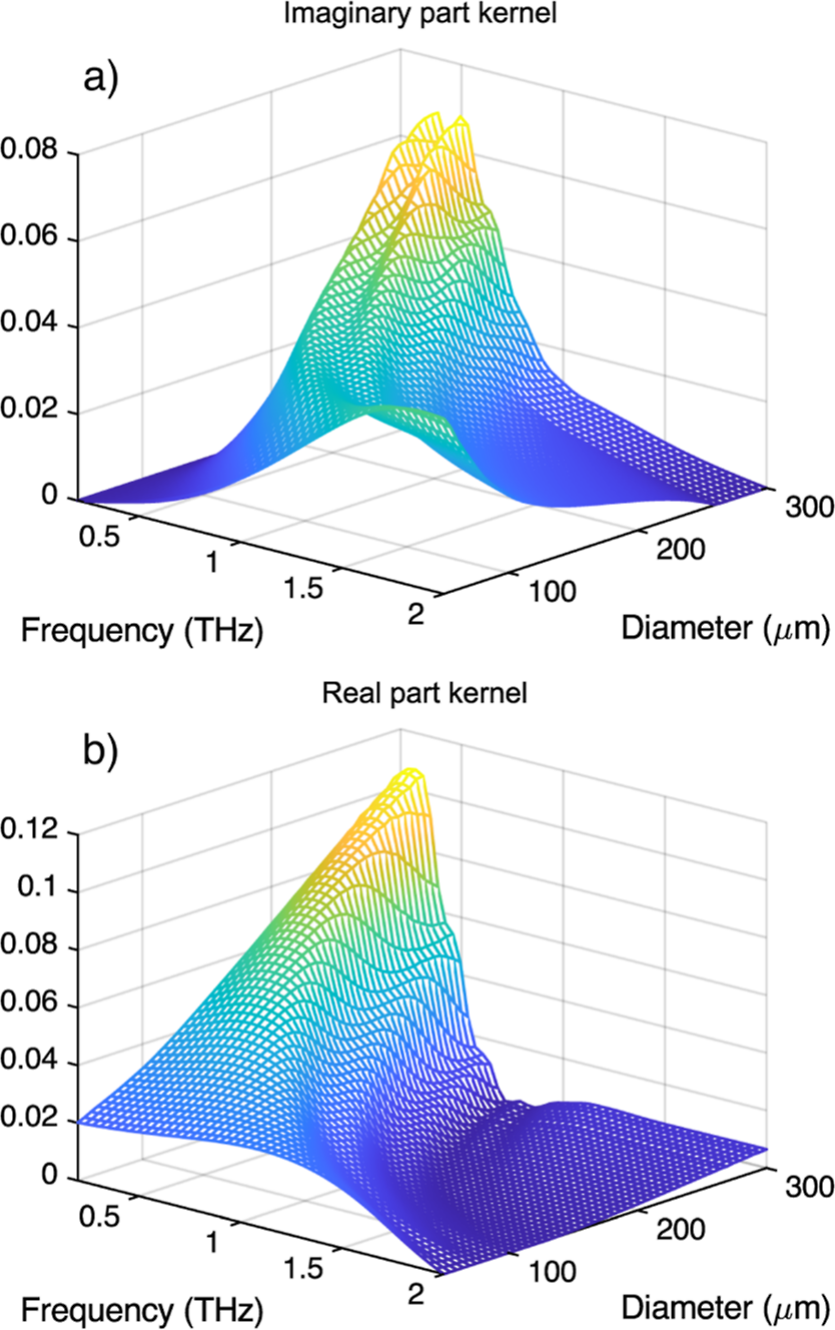
Imaginary (a) and real (b) kernels used for iterative inversion corresponding to a system with a relative refractive index of 1.88 + *i*0.24 × ν[THz].

These kernels need only to exhibit the functional form of the extinction and refractive index for each (ν, *D*) pair. Because of this, the particle density was chosen to be *N*_0_ = 6ϕ/(*piD*_mid_), where ϕ is the volume fraction of particles in the medium and *D*_mid_ is the mid value of the diameter vector used for inversion. A graphical representation of the shape of the kernels is shown in Figure 2.

The choice of an initial guess *f*_0_^*j*^ for the iterative process has been discussed^45^ and, instead of the more usual choice of a uniform distribution, a better choice seems to be a power law function centered in the range of diameters of choice. This is due to the iterative factor having a tendency to overestimate the value of the solution for small sizes. In light of this, we employed an initial solution of the form *f*_0_ = *D*^8^/max(*D*^8^) in order to ensure a value of unity at the largest diameter.

As far as the stopping criterion is concerned, for each iteration, the relative increment from the last iteration was calculated as follows
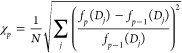
8

This quantity gives an idea of the speed of convergence of the algorithm, and a tolerance value can be set below which changes in the solution are negligible. In the following, we stopped the iteration when a value of 10^–4^ was reached.

In eq 7, each of the two components of the iterative factor, i.e., extinction and refractive index, is weighted by means of parameter α. This makes it possible to control the behavior of the iterative method to balance the impact of extinction or refractive index information. When the parameter is 0, only refractive index is considered. When the parameter value is 1, only extinction data are considered and thus the iterative method will more closely follow the TM approach.

### THz Spectrometer

**Figure 3:**
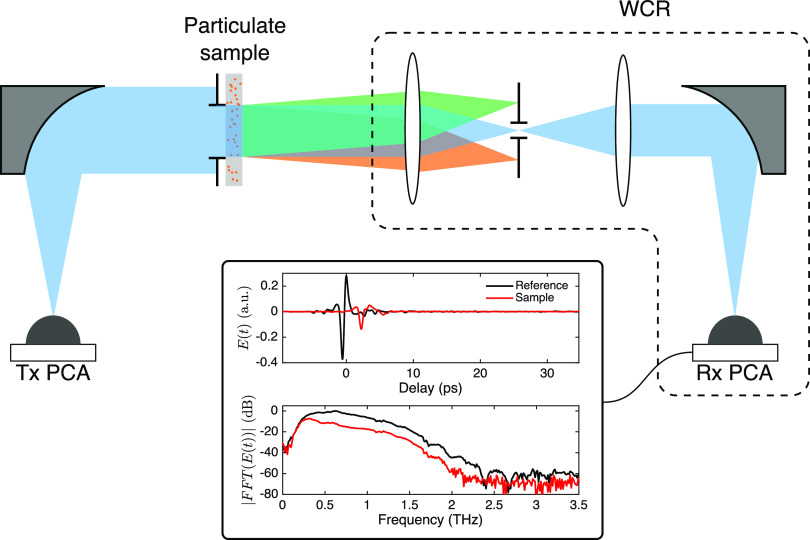
Block diagram of the experimental THz setup used in our measurements. The THz beam generated at the transmitter photoconductive antena (Tx PCA) is collimated by an off-axis parabolic mirror. The sample is positioned after a diaphragm that regulates beam width, and then, two lenses and a pinhole aperture are positioned in a “well collimated radiometer” configuration in order to restrict the angular acceptance of the receiver. This ensures an accurate measurement of the extinction caused by the sample. The center box shows the pulse amplitude and spectral magnitude of a sample–reference pair of measurements.

For the experimental demonstration, an in-house-built THz spectrometer based on fiber pig-tailed photoconductive switches at 1550 nm and a usable bandwidth of 2 THz was employed. Delay mapping was performed with a voice coil optical delay line with an approximate range of 50 ps at a speed of 5 traces/s. The incoming THz signal was digitized at 100 KS/s and each THz measurement was the result of averaging over 250 time traces. The THz beam was collimated and refocused with a pair of off-axis parabolic mirrors. As can be seen in Figure 3 two diaphragm irises were employed. The first one sets the diameter of the beam so that no light falls outside the sample diameter. The second one limits the acceptance angle of the sampled beam in what is known as a “well collimated radiometer” (WCR). This configuration minimizes the amount of stray light that enters the receiver coming from the scattering centers^46^ and thus ensures that the measured quantity in a sample/reference experiment corresponds to the macroscopic extinction created by the heterogeneous medium in the forward direction and the phase resultant from far field interference between incident and scattered radiation. In all our measurements, the semiangle of acceptance was kept at 2°.

The optical parameters of the agglomerates were obtained in the following manner: first, the sample and the spectrometer were placed in a sealed enclosure that was purged with dry air to a relative humidity of around 15%. Then, a 50 ps pulse trace is obtained with (*E*_sam_) and without the sample (*E*_ref_) a total of 5 times. Between each measurement, the sample was rotated in its holder in order to average several configurations of the heterogeneous medium with the same characteristics. After acquisition, the frequency domain versions of the sample and reference measurements (*Ẽ*_sam_ and *Ẽ*_ref_) are obtained via the fast Fourier transform. From here, the extinction and refractive index of the sample can be calculated using eqs 9 and 10
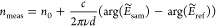
9
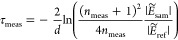
10where arg() is the argument operator, *d* is the thickness of the heterogeneous medium, *n*_0_ is the refractive index of the air, *c* is the speed of light in a vacuum, and ν is the working frequency.

### Sample Preparation

Samples were tablets made from very finely ground poly(tetrafluoroethylene) (PTFE, Teflon) particles (approximately 3 μm) that serve as a continuous background medium when pressed. The sample particles were spherical soda lime glass beads belonging to two classes of the NIST-traceable 9000 series provided by Duke Standards and calibrated by optical microscopy. Its tabulated size distributions have a mean diameter of 97 μm with a standard deviation of 3.9 μm and a mean diameter of 233 μm with a standard deviation of 8.5 μm (Figure 6 bottom row). Both materials were mixed and pressed into tablets 22 mm in diameter and around 2 mm thick using 6 tons of force. Three samples were created, two with a 5% volume fraction of each particle size and a third one mixing 3% of the 97 μm size and 5% of the 233 μm size. This choice was made to validate our particle sizing technique with both monodisperse and polydisperse samples.

Since both matrix and particulate materials are slightly absorbent, their refractive index must be carefully tabulated for the inverse model. Because of this, we made a tablet containing only pressed PTFE and its optical properties were characterized. For the soda lime glass, composition values given by the manufacturer matched those of Schott B 270 glass and the complex refractive index for this material was taken from an experimental study by Naftaly and Miles^47^ (see the Supporting Information).

## Results

### Simulation Validation

**Figure 4:**
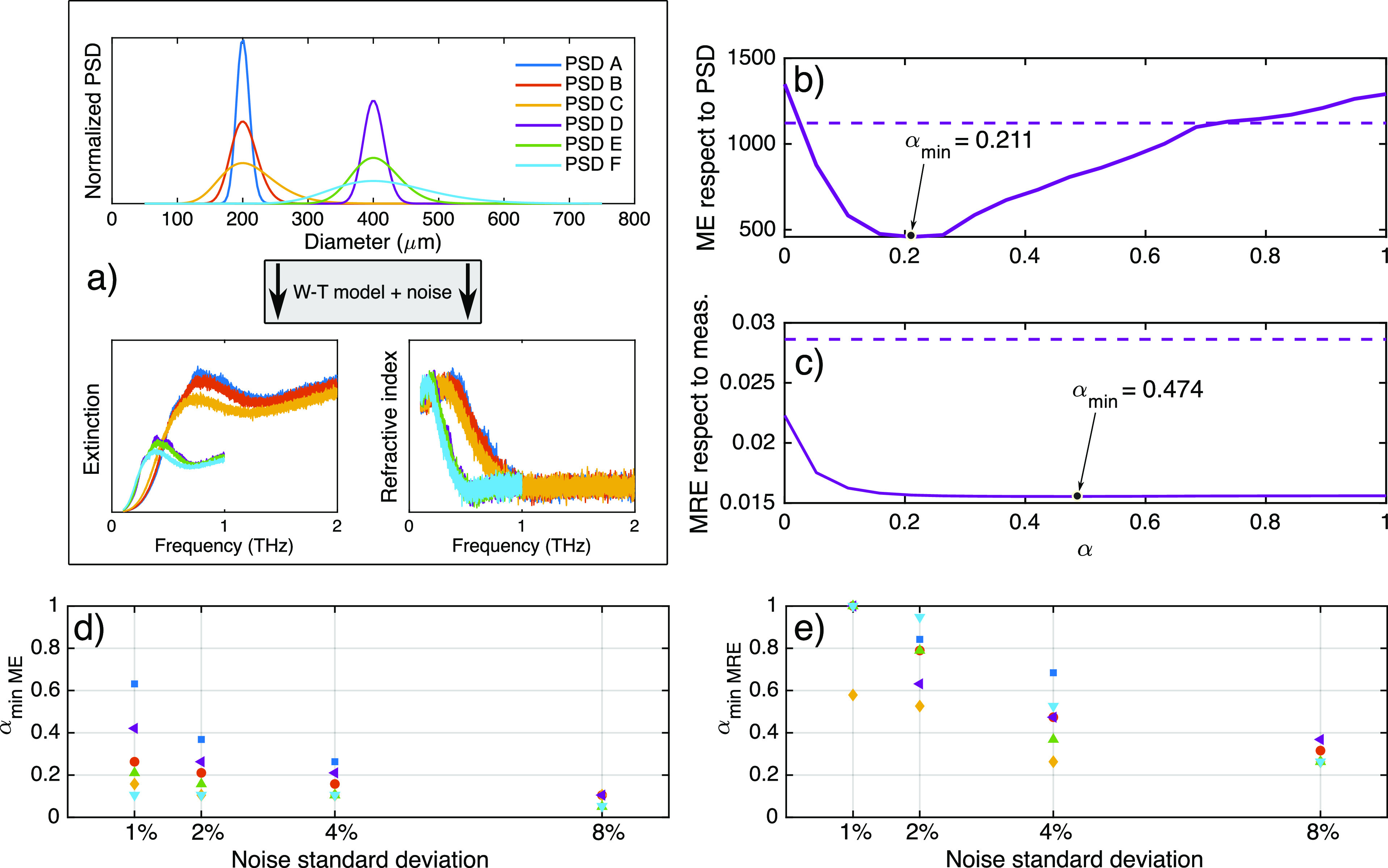
(a) Creation of synthetic optical parameters starting from a selection of PSDs that are fed to the WT model and then corrupted with noise. (b,c) Performance metrics of the inversion of artificial measurements of PSD D and a noise standard deviation of 4% for extinction and 0.4% for refractive index versus different α values. (b) Shows the average error between recovered PSD and original PSD and (c) shows the average of relative errors between calculated and synthetic measurement of the optical parameters. Dotted line shows the error value when using the Twomey method. (d,e) Show the position of the minimum α for the ME and MRE figures, respectively, and for different noise strengths.

In order to evaluate the performance of the new inversion method with respect to the α parameter, synthetic data were generated by running the forward model based on the WT formula with several PSDs (see Figure 4a) and a given refractive index as input. These data were generated from a set of diameters much denser than the ones employed later in inversion in order to avoid what is known as inverse crime,^42^ which leads to excessively optimistic results stemming from having identical forward and inverse models. The synthetic optical parameters were generated in a set of frequency points close to the realistic range found in the THz-TDS experiments. For PSDs A-C, the maximum frequency was set to 2 THz and for PSDs D-F, the maximum frequency was 1 THz. Since the frequency features of the optical parameters scale with mean particle size, these limits ensured that the Mie resonances present in extinction and refractive index occupy a similar fraction of the frequency range. We found that this asymmetry was necessary in order to ensure the stability of the inversion procedure, so in a realistic scenario, one must choose the maximum frequency range available but not extend it too much beyond the Mie resonances.

After synthetic extinction and refractive index measurements were obtained, these were corrupted with different levels of white noise characterized by a relative standard deviation ranging from 1 to 8%. Based on our previous observations with THz-TDS systems, we considered more realistic to give refractive index data an order of magnitude less relative error than extinction data, i.e., 0.1–0.8%. A justification for this is provided in the Supporting Information. We repeated this procedure for 50 different noise seeds and then performed inversion of each set of data.

Results of the inversion of synthetic data show noticeable variation with α. In order to asses this variation, the mean error (ME) with respect to the original PSD was calculated as follows
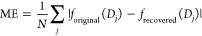
11where *f*_original_ and *f*_recovered_ are the normalized versions of the seed PSD for the simulations and the solution after inversion, respectively. In all cases, the ME versus α curves (Figure 4b) present a minimum value between 0 and 1. These minima are plotted in Figure 4d for each noise level. Also for comparison, the synthetic data were inverted with the TM described in eq 6. The ME was also calculated for the TM and is shown in the dotted line. In all cases, the minimum value of α using the FFTM produces a lower error than the TM, which demonstrates that the addition of a term containing refractive index data benefits the inversion process. Obviously, in a practical scenario, the choice of the α value cannot be made this way. Because of this, the following blind metric was used to decide what α value to employ.
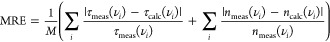
12

**Figure 5:**
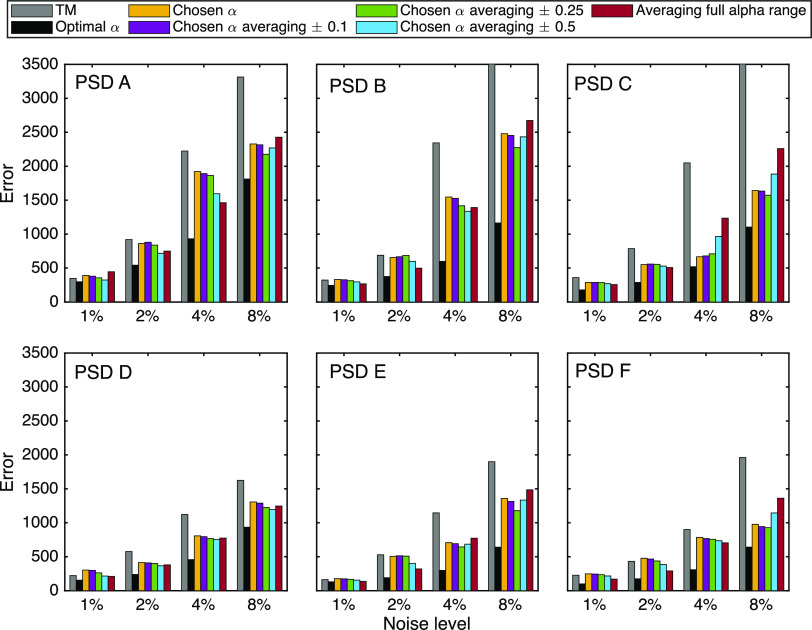
ME figure shows the simulated measurements and each level of added noise. Black bars show the error of the alpha value with lowest ME. For comparison, gray bars show the error obtained with the TM. Colored bars show different levels of averaging around the best alpha following the MRE criterion.

This mean relative error (MRE) corresponds to the sum of the mean relative residuals of the extinction and refractive index data. In Figure 4c,e, MRE is found to have minimal α values close to the true optimal and thus it is likely a good predictor of the best value to use without resorting to a priori information. However, MRE curves often present a flat plateau at high α values that could imply a lack of the decision power of the criterion. In these cases, we observed that averaging the solutions for several α values adjacent to the minimum MRE gives a better estimate of the true PSD. Figure 5 shows the ME compared to the error using the TM for different averaging ranges and all of the simulated PSDs. In black, the error when selecting the optimal α value with the minimum ME can be seen to always be smaller. When using the realistic MRE criterion, however, the error is usually between the optimal and the TM. The error improvement tends to be smaller for the synthetic measurements with lowest noise added (1–2%) which represent the least realistic situations. For higher noise (4–8%), the improvement is much greater, meaning a better inversion performance than the TM. When considering averages of solutions for α values around the MRE minimum, a range of 0.25 seems to be the best compromise among the tested PSDs.

Additionally, there appears to be a correlation between lower α values (more weight toward the refractive index term) and a smoothing of the retrieved PSD. This is more pronounced with increasing levels of noise, which tend to have the minimum MRE point at a lower α value (see Figure 4d,e). In turn, this could be related to the fact that narrower PSDs such as PSD A and D had a higher optimum α than broader (and thus smoother) PSDs such as PSD C and F.

### Experimental Validation

**Figure 6:**
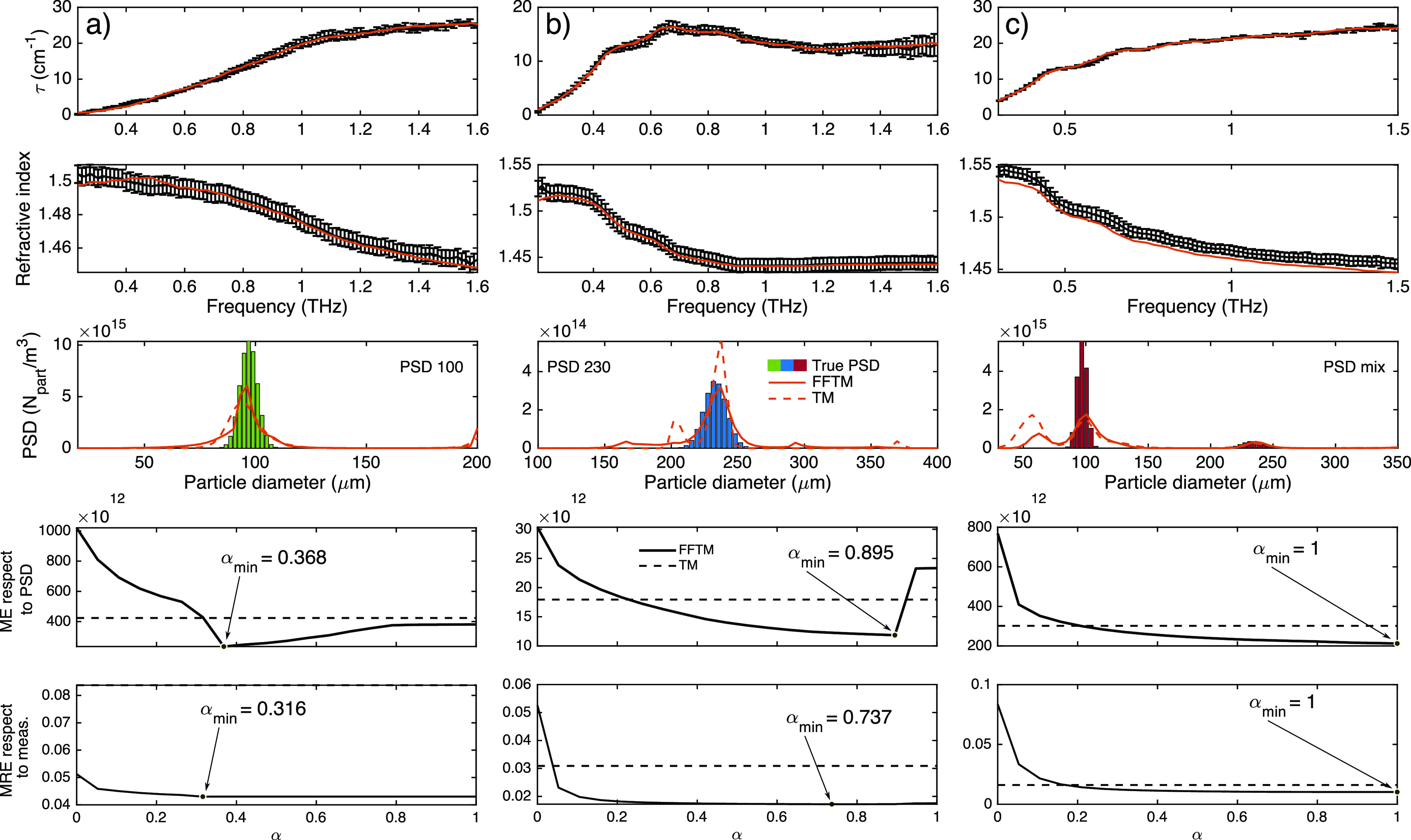
Two top rows show the extinction and refractive index of three granulated samples made out of pressed PTFE powder and glass spheres with different PSDs. Volume concentrations of glass for columns (a,b) were 5%, for column (c) it was a mixture of 3% of the PSD 100 and a 5% of the PSD 230 spheres. In orange are the reconstructed optical parameters obtained through the forward model using the PSD solutions in the middle row. Inversion was performed using an α value with the minimum value of the MRE metric, and the final PSD comes from averaging the solutions in a range of ±0.25.

The proposed full field particle sizing technique was experimentally validated by performing THz-TDS measurements of the extinction and refractive index of tablets made by pressing powder mixed with spherical particles (see Sample Preparation). Results of this can be seen in Figure 6, where the first and second rows show experimental extinction and refractive indexes in black corresponding to τ_meas_ and *n*_meas_ averaged over five repetitions for each sample. In orange, are the values obtained from the measurements after the retrieved PSD is employed to compute τ_calc_ and *n*_calc_ following eq 3. The results show a good agreement with the true PSD and that FFTM outperforms the conventional intensity-based TM. The middle row shows, in bars, the original PSDs given by the manufacturer. The size of these PSDs is calculated according to the volume fraction values that were used for the fabrication of the samples. Also shown in those plots are the retrieved PSD after the last iteration of the recursive method. For these inversions, the number of frequencies and diameters used was 60 and the value of α was that with the minimum value of the MRE metric (Figure 6 bottom row) and an averaging of ±0.25 was performed around that value. For comparison, the inversion solution and calculated optical parameters with the Twomey method are shown in discontinuous line.

As with the inversion of synthetic measurements in simulation validation, we performed inversion with several values of α and compared the results with the PSD given by the manufacturer. As can be seen from ME values in the last row of Figure 6, better results are achieved with our modified inversion method, especially for the sample with a larger mean particle size.

## Discussion

Particle sizing using both extinction and refractive index curves shows promising results for a more precise PSD determination. Simulation and experimental results show that the experimental error decreases. Simulations predict a maximum error improvement of up to 65% and experimentally, a 32% decrease was achieved with a blind determination of α.

The acquisition of the refractive index does not necessarily need to come from direct measurements as demonstrated in the present work by the use of THz-TDS. Refractive index data could be indirectly computed from amplitude measurements and an appropriate application of the Kramers–Kronig (KK) relations, as is common practice in reflectance spectroscopy.^48^ This would be an ideal situation that would benefit from the additional numerical stability of the FFTM and would require less experimental complexity than a combined amplitude and phase measuring setup. However, in practice, KK phase retrieval needs a broad range of measuring frequencies in order to fully capture the relevant resonant peaks.^49^ This situation is rarely met in forward scattering measurements, where scattering spectra typically present broad features that are usually measured at the border between Rayleigh and Mie regimes.

As seen from the experimental results, PSDs with a higher mean diameter present a lower error. This was expected due to the fact that the maximum of the extinction curve is located at a lower frequency for PSD 230 and thus more of the complex scattering features are located inside the usable frequency band. This effect is not observed as clearly in the inversion of simulated measurements because the frequency range was adjusted, depending on the mean particle diameter, to always fill the same proportion of the characteristic Mie resonances.

In general, the range of sizes will be greatly dependent on the measurable frequency band and refractive index contrast. Usually a good way of estimating the minimum range is locating the size for which the maximum of the Mie extinction coefficient just barely makes it into the measurement frequency range. For the cases presented in this work, the refractive index contrast is about 1.85, the maximum measurable frequency is around 1.5 THz and the Mie resonant peak is at a size parameter of 2.3. This would set the minimum measurable diameter at 146 μm and using our technique we managed to invert, albeit with some error, a PSD with a mean diameter of 100 μm. This favorable result at small particle sizes suggests that the wide range of frequency components available in THz-TDS, much greater that traditional sizing techniques, helps overcoming the theoretical limit of sizing techniques based on spectral scattering.

As we observed with the inversion of synthetic measurements, the iterative inversion method becomes unstable when a large portion of the spectrum falls beyond the characteristic Mie resonances. This points toward the shortening of the frequency range for large particle diameters, and experimentally, the need arises for a finer spectral resolution that can properly resolve the smaller spectral features. Because of this, the maximum particle size would instead be determined by the capability of the THz spectrometer to resolve fine spectral details, which become smaller as the mean particle diameter increases.

Nevertheless, the relatively large central wavelength allows for the measurement of a range of particle sizes that are beyond the scope of laser-based techniques. Since, at visible wavelengths, Fresnel diffraction is used as the forward model, only opaque particles can be measured so that refraction inside the particles does not interfere with the far field diffraction patterns.^43^ This makes THz based sizing a promising alternative to measuring industrially relevant aggregated samples in the upper range of particle sizes.

Refractive index contrast acts as a limiting factor in a similar way as particle size. A lower refractive index contrast will make the Mie resonances appear at higher frequencies, and thus, a larger maximum frequency will be needed for the same particle size. On the contrary, a large refractive index contrast, as can be the case for plasmonic nanoparticles, will increase the amount of spectral oscillations and lower the frequency of the resonances. This will thus require a higher spectral resolution, as stated above.

Another factor to take into account is the concentration of the samples. The highest measurable concentration depends mainly on the range of validity of the scattering model. For this work, no more than a 10% volume fraction would be measurable since the WT theory does not properly account for dependent scattering interactions. A more extensive study of the errors arising from this can be found in the Supporting Information. Additionally, the dynamic range of the measuring instrument is also responsible for the concentration limits. A very dense sample would drastically increase the extinction, possibly equating the received signal to the noise floor. A very dilute sample, on the other hand, would not create enough spectral features to distinguish the optical parameters from those of the surrounding medium, increasing the inversion error.

## Conclusions

The feasibility of coherent particle sizing by inversion of extinction and refractive index measurements was presented and demonstrated. The well-known iterative Twomey method has been expanded to include refractive index data. Through the creation of synthetic data, we performed a careful analysis of the influence of changing the weight between both kinds of data sets, elaborated a criterion for choosing an appropriate value, albeit not yet in a fully optimal manner, and concluded that combining amplitude and phase information improves the quality of the inverted PSD in noisy scenarios. We validated our method by measuring pressed tablets of PTFE and glass beads and inverting the PSD of the spheres inside. Monomodal and bimodal distributions were inverted without any a priori knowledge. The method does not require any calibration.

Future improvements of the technique could involve a better criterion for the blind estimation of the α parameter that provides a solution closer to the one with the lowest error. Also, machine learning optimization methods are quickly gaining popularity for the task of inversion of extinction data and we intend to apply these algorithms to full-field particle sizing.

The method has been realized by exploiting THz waves and can be regarded as the first quantitative approach to particle sizing using this band. This has potential for measuring agglomerates that are optically opaque, allowing the characterization of industrially relevant compounds in solid form after assembly of the product and even within packages. In addition, this approach is not restricted to the THz band but could be applied to any experimental field where the acquisition of amplitude and phase information is available such as microwave or ultrasound spectroscopy.

## Supporting Information Available

Optical constants used for the inversion of experimental measurements, justification of the noise levels used in the simulated measurements, and a comment on the limits of the scattering model employed for higher volume fractions (PDF)

## Notes

The authors own a patent on the method for improved PSD characterization described in this report.
