# Supplementary material for: Particle Size Inversion from Spectrally Resolved Full-Field Forward Scattering
Source: Anal Chem. 2023 Oct 17;95(43):15994–6003. doi: 10.1021/acs.analchem.3c03178 (PMC10620756; doi:10.1021/acs.analchem.3c03178)
Supplement: Supplementary file 1 — ac3c03178_si_001.pdf [file ac3c03178_si_001.pdf]

Supporting information

Particle size inversion from spectrally resolved full-field forward scattering

Miguel Ángel Báez-Chorro[1] and Borja Vidal[1]

[1]*Nanophotonics Technology Center, Universitat Politècnica de València, Valencia, 46022, Spain*

Contents

1 Optical constants of experimental samples **1**

2 Relative errors of THz optical parameters **1**

3 Limits of volume fraction for inversion with the WT model **2**

1 Optical constants of experimental samples

The optical parameters of the medium and particulate material used in the experimental samples are shown in Fig S1. We measured the matrix medium from a tablet made of the same PTFE powder employed in making the granulated samples. We used the same THz spectrometer, compression force and dimensions. The particulate medium was characterized with a refractive index and extinction from reference [1] corresponding to the glass B270.

2 Relative errors of THz optical parameters

In the main manuscript, we test the inversion of PSD by first creating synthetic measurement data that are corrupted with Gaussian noise in order to better approximate a realistic measurement. The amount of noise

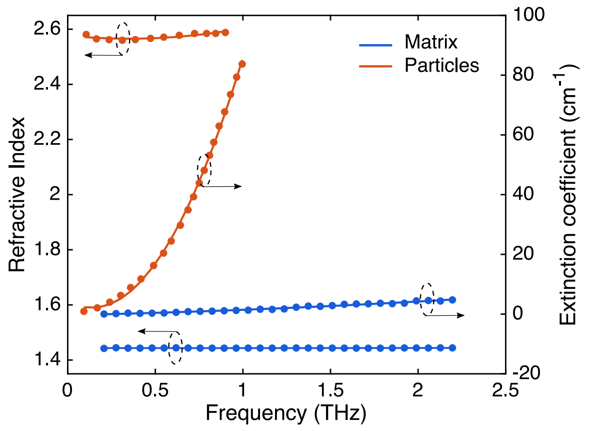

Figure S1: Optical parameters of particle and matrix medium. Medium material is PTFE polymer and particles are made of B270 glass. Dots are experimental points and continuous lines are a best fit to a second degree polynomial.

added to each frequency point follows a distribution with a standard deviation given by a set of percentages of the value of the optical parameter (1%, 2%, 4% and 8% for extinction and 0.1%, 0.2%, 0.4% and 0.8% for the refractive index). This choice is motivated by the errors observed in our experimental measurements during the development of the method. An example of typical measurements performed with the same instrument described in section THz Spectrometer of the main manuscript is shown in Fig. S2. The error bars in that figure come from 5 repetitions of sample placement inside the THz spectrometer. As can be seen in the bottom graphs, relative errors across the relevant frequency range are in the order used for the simulations and typically are higher than 2% for extinction and 0.2% for refractive index.

3 Limits of volume fraction for inversion with the WT model

In this work, we employ the WT approximation as the forward model for inversion. Since this is a low order approximation of multiple scattering, an analysis of the limits of inversion by this method is in order. To test this, we simulate a set of spectrally resolved optical parameters using QCA theory for a given particle size, relative refractive index and volume fractions of 2.5%, 5% and 10%. Then, we employ the proposed full-field inversion method that uses the WT forward model to derive particle size from scattering simulations. PSDs retrieved this way can be seen in Fig. S3. As was expected, inversion starts to lose accuracy beyond a 5% volume fraction and is rather inaccurate for 10%, where not only a shift in the mean diameter occurs but an additional peak appears at low diameters.

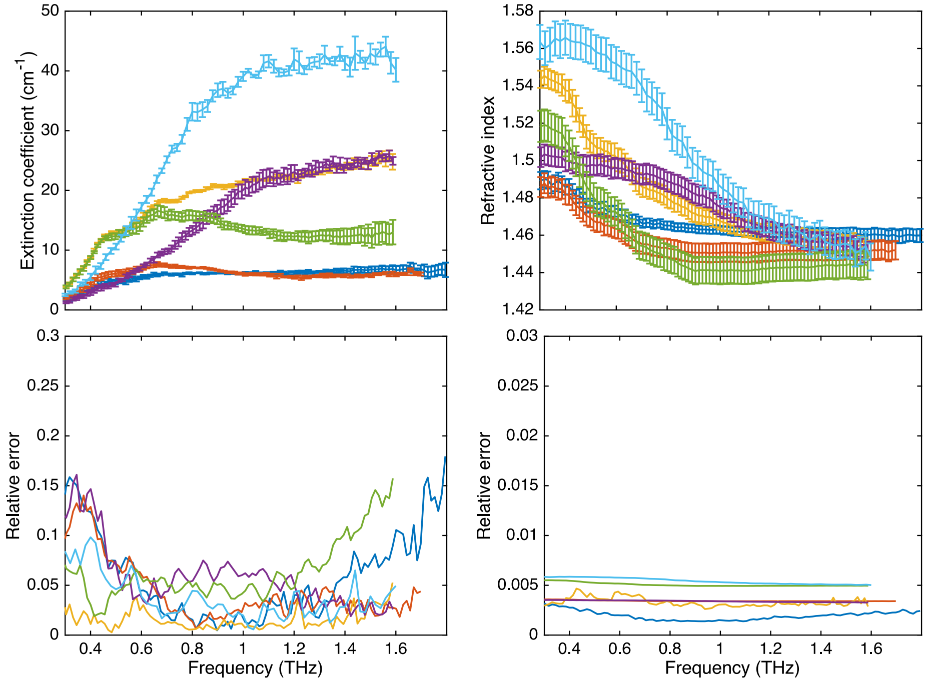

Figure S2: Optical parameters of several sample measurements and their respective relative errors. Experimental error bars come from 5 repetitions of sample placement in the same spectrometer used in the main text.

References

[1] M. Naftaly and R. E. Miles. Terahertz time-domain spectroscopy of silicate glasses and the relationship to material properties. *Journal of Applied Physics*, 102(4):043517, 2007.

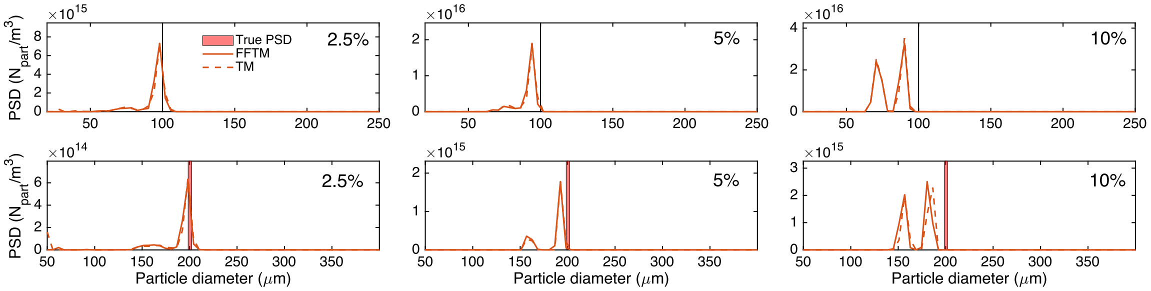

Figure S3: Results of inversion of artificial simulation data created from QCA and inverted using a scattering model based on the WT equation.
